# Cases from the Surgical Clinic of the Philadelphia Hospital

**Published:** 1860-05

**Authors:** Emil Fischer

**Affiliations:** Philadelphia


### Art. I.—*Cases, with Remarks, from the Surgical Clinic of the Philadelphia Hospital.*—Service of Professor Gross.

Reported by Emil Fischer, M.D., of Philadelphia.

I. *Three Cases of Incontinence of Urine treated by the Application of the Actual Cautery to the Lumbar Region.—Case* 1.—John H., thirty-five years old, a carpenter, was admitted into the surgical wards of the Philadelphia Hospital, in January, 1860, on account of being unable to retain the contents of his bladder. He states that, about two years ago, while working at his trade, he fell from a building, striking on his back, and receiving a severe contusion across the lumbo-sacral region. The injury was followed by loss of consciousness, which lasted for an hour, after which he gradually recovered from the shock, retaining the perfect use of his limbs, and suffering from no bad symptoms. About seven or eight days after the accident, he noticed, for the first time, that he could not control his bladder as well as usual, but that the water dribbled away after having accumulated for a short time in the bladder; since then the difficulty had been gradually increasing. At the time of his admission the patient suffered from complete incontinence, the water dribbling off constantly, except at night, or whenever he assumed the recumbent position; it was then retained for two or three hours at a time, but ran off as soon as he rose from the bed. He did not complain of scalding, nor of pain in the region of the bladder or in the groins, but suffered occasionally from pain in his back. His general health has always been good, and the action of his bowels regular. On examining the case with the catheter, Professor Gross convinced himself that there was no foreign body present in the bladder, but thought that he could distinguish a fasciculated, hypertophied condition of that organ. The history and nature of the case led him to the conclusion that the proximate cause of the incontinence was paralysis of the bladder.

As the patient had already been subjected to various methods of treatment without receiving any real benefit, and as the other remedies usually employed in cases of this kind did not promise to be of much avail, Professor Gross proposed to make an issue in the upper portion of the lumbar region, which might furnish an abundant discharge and a clean surface for the application of remedies. The actual cautery was consequently applied on the seventh of February, the patient being under the influence of chloroform. The cauterized parts were dressed at first with cold water, and afterwards with an emollient poultice. An issue of the size of half the palm of the hand was thus produced, and a free discharge maintained by the occasional use of savine cerate. The improvement following this treatment was rapid and really astonishing, proving the great advantage of this kind of issue over all others, and the powerful impression it exerts both upon the part and upon the system. On the fourteenth of February, when Professor Gross brought the case again before his class, the patient stated that his water did not dribble off any more when he was walking about, and that he could retain it for several hours at a time, no matter what position he assumed. On examination, his urine was found to be acid, and to contain a large proportion of mucus. He was now put on the use of bicarbonate of soda and tincture of belladonna, with which he continued until the thirty-first of March, when he was almost entirely well.

*Case* 2.—Hannah D., aged about twenty-five years, of temperate habits, and robust frame, entered the surgical wards of the Philadelphia Hospital, in November, 1859. At the time of her admission she complained of being unable to retain her urine, it being voided involuntarily almost as fast as secreted. With the exception of this affection, which, on account of its inconvenience and disagreeable character, proved a source of great annoyance to her, she was free from any complaint. She suffered from no particular pain; her general health was undisturbed, and had always been so, according to her recollection, since childhood; her bowels acted well; her appetite was excellent; her menstrual functions were performed with great regularity; and she had never suffered from any uterine affection. The incontinence of urine came on without any assignable cause; the patient could not recollect that she had ever had a fall, been hurt in any manner, or had suffered from any serious symptoms: the uterus was in its natural position. For some time after her admission she was put successively on the use of various remedies without being benefited by them; the urine continued constantly dribbling, causing her linen to be always wet, and rendering it necessary for her to remain continually in bed. Dr. John W. Lodge, one of the resident surgeons of the hospital, inspected her urine carefully with the microscope, and found a copious deposit of phosphates with numerous epithelial casts and mucous corpuscles, the fluid being at the first examination alkaline, and subsequently neutral. The prescribed remedies not producing any appreciable effect, Professor Gross subjected the patient to a more rigid examination, during which he found that on making pressure upon the lumbar portion of the vertebral column, the woman complained of exceedingly acute and diffused pain. He consequently determined to apply the actual cautery over this point. In the early part of January, 1860, a large eschar was accordingly made with the iron at white heat, immediately over the lumbar region; the cauterized parts were dressed with cold water until the detachment of the slough; afterwards the sore was kept open, covered with a poultice, and the discharge promoted with savine ointment. The success following the treatment was very marked; the patient continued to improve rapidly and permanently, and at the end of five or six weeks was completely well, not being compelled to empty her bladder more than three or four times in the twenty-four hours. The issue was kept discharging for some time afterwards, and then allowed to granulate and heal. After the marked improvement of the symptoms the urine was examined again under the microscope, and was found natural, except an undue deposit of mucus and epithelium.

The patient continues perfectly well regarding the incontinence, but is now suffering under an attack of Bright's disease, the symptoms of which commenced to show themselves early in March. The albuminuria presents no facts of particular interest, but appears to be merely an intercurrent disease, the woman having been healthy up to the time of its appearance. Her urine has lately been subjected to repeated examinations, and was found to coagulate on the application of heat and nitric acid, and presented under the microscope numerous casts of the uriniferous tubules. Under the use of an invigorating treatment and the occasional exhibition of a little elaterium, her health is improving, and favorable hopes are entertained of her ultimate recovery.

*Case* 3.—James B., nineteen years of age, of dark complexion, robust frame, and apparently full habit, was admitted into the surgical wards of the Philadelphia Hospital, in the early part of January, 1860, on account of nocturnal enuresis. He has suffered from the disease for eight years, wetting his bed once or twice every night, but having complete control over his bladder when awake. Although having nearly approached to the years of manhood, he has been kept from learning any trade, as his different employers always discharged him as soon as they found out that he was laboring under so loathsome an affection. On catechizing the patient rigidly, it was ascertained that he was given to masturbation, a circumstance which served to explain the origin and the obstinate character of the disease. Dr. John W. Lodge, who examined his urine microscopically, found it to contain an abundance of uric acid crystals. Soon after his admission, the patient was put on the use of strychnine and cantharides, but these remedies producing no effect, Professor Gross concluded to apply the nitrate of silver, by means of the porte caustique, to the prostatic region of the urethra, which was accordingly done in the latter part of January, before the clinical class. The patient suffering from some pains after the application, he was put on the use of demulcent drinks, with bicarbonate of soda and paregoric. The cauterization was followed apparently by success; the youth could hold his water completely for several nights in succession; but the improvement was not lasting, and in less than two weeks a relapse took place, the patient wetting his bed as regularly as before. In the early part of February the cauterization was repeated, and the patient was ordered to take fifteen grains of bromide of potassium, with a tenth of a grain of sulphate of morphia, three times daily; but still the affection continued unchanged with annoying obstinacy. Encouraged by the great success which followed the application of the hot iron in the two cases reported above, Prof. Gross determined to try the same remedy in this instance. In the latter part of February the actual cautery was accordingly applied in the upper part of the lumbar region, the patient having been put under the influence of chloroform, and the issue thus produced was kept open by the use of savine cerate. The application was promptly followed by decided improvement. The patient wet his bed only twice during the six weeks succeeding the operation, and then probably in consequence of having committed some indiscretion in his diet. His urine, which was examined by Dr. Lodge about a week after the application of the cautery, was found to contain under the microscope but few uric acid crystals, but more oxalate of lime and epithelial scales; its reaction was acid; it did not exhibit any perceptible sediment, but had an abundance of mucus mixed with it. At present, in the early part of April, the cure can be considered as almost perfect; the patient, on waking up in the morning, finding his bed always clean and dry.

*Clinical Remarks.*—In presenting the cases reported above, to his class, Professor Gross made the following observations: Incontinence, or involuntary discharge of urine, is an affection liable to occur at any period of life, and may be either partial or complete, temporary or permanent. Some writers distinguish an active incontinence, where the necessity of voiding the urine is felt, but the will exercises no control over the discharge; a passive one, without any perception of this necessity; and a spasmodic one, resulting from great irritability of the neck of the bladder.

Incontinence, strictly so called, results from a condition of the bladder and urethra the reverse of that of retention. For, while retention is produced whenever the action of the bladder is weakened and the resistance in the neck of the bladder and urethra is increased, incontinence, on the contrary, originates either from the expelling power of the bladder being augmented while the resistance in the urethra is not proportionally increased, or from the resistance being lessened while the expelling force continues the same. Incontinence may, however, be associated with retention. This is the case when the disease depends upon weakness and paralysis of the bladder. Owing to the fact that the sphincter muscle generally retains some contractile power, more or less of the urine is apt to accumulate in the bladder, while the rest gradually passes off, leading thus to a belief, on the part of the practitioner, that the case is one purely of incontinence, when, in fact, it is one both of incontinence and retention. An illustration of incontinence from paralysis of the bladder will be found in the first of the cases reported above, in which the disease followed a severe fall upon the back. Thus, any direct injury, as a serious contusion of the abdomen, pelvis, or perineum, may induce paralysis of the bladder, or of this viscus and the urethra, and, consequently, incontinence. Sometimes the affection is owing to some disorder of the sacro-lumbar nerves. In the latter months of pregnancy, as well as during protracted parturition, the neck of the bladder is sometimes paralyzed by the pressure of the child's head, and the incontinence thus produced may last a long time. This affection is occasionally met with in fever, in concussion of the brain, in injury of the spinal marrow, in hysteria, epilepsy, and other nervous diseases. Old men who have led irregular and dissolute lives, and who have labored long under disease of the urinary passages, are very prone to suffer from incontinence of urine.

In the treatment of incontinence from paralysis, our remedies must be chiefly addressed to the invigoration of the nervous system. For this purpose, after having cleared out the bowels and corrected the secretions, the patient is put on the use of strychnia, either alone or combined with some mild tonic, such as extract of cinchona and sulphate of iron. Cantharides may also be advantageously given, especially if they be carried to the extent of slight strangury. The diet should be light, and the patient should make frequent use of the cold shower-bath, followed by dry frictions. Counter-irritation is kept up in the sacro-lumbar region either by a succession of blisters, tartar emetic ointment, the moxa, or, in obstinate cases, by that most powerful counter-irritant, the actual cautery.

Incontinence may arise from a *morbid sensibility* of the neck of the bladder, or of the entire organ. A peculiar variety of this form of the disease is the nocturnal incontinence which occurs in *young subjects*, and of which the third of our cases affords an example. It is most common in children before the period of puberty, and often begins at a very early age. The discharge, which may take place twice or even thrice during the night, is sometimes effected under the influence of the will or a dream, but in general it is strictly involuntary. When it becomes habitual, as in fact it usually does, it may last for years, and be even prolonged into advanced life, though in most cases it gradually disappears on the approach of adolescence. It is promoted by the use of fluids, by exposure to cold, and by sleeping on the back, a posture which is favorable to the accumulation of urine in the morbidly sensitive portion of the bladder.

The pathology of this affection consists in an exaltation of the natural sensibility of the mucous membrane of the neck of the bladder, unaccompanied, in many cases, by any appreciable change of structure. Sometimes there is slight thickening of the part, and occasionally the affected surface is somewhat inflamed. In protracted cases, there may be hypertrophy of the prostate gland, though never to any considerable extent. The sphincter of the bladder is easily relaxed, and yields to the most trifling impulse; hence the urine often flows off even when there is no fullness or distention of the organ.

Morbid irritability of the neck of the bladder, or of the entire organ, giving rise to incontinence of urine, may be excited by the acid character of the urine, or by sympathy with the kidneys, rectum, anus, vagina, or uterus. Worms in the lower bowel, hemorrhoidal tumors, and fissure of the anus, are often attended with incontinence. Masturbation or inordinate sexual indulgence, by establishing a morbid sensibility of the mucous membrane of the neck of the bladder or commencement of the urethra, may be followed by the same result. In most of these instances, the incontinence is incomplete.

In the treatment of incontinence arising from morbid sensibility of the bladder or its neck, particular inquiry should be made into the nature of the exciting cause, the removal of which is of paramount importance. In that variety of the affection which is met with in boys and girls, the cure may be greatly expedited by proper attention to the diet, which should always be bland and unirritating. Late suppers are avoided, and the patient must abstain entirely from drinks for several hours before going to bed. During the night, he is to be waked two or three times for the purpose of emptying his bladder, and this practice is to be persisted in for weeks, and even months, until the disagreeable habit is broken up. During all this time, as well as indeed for a long period afterwards, the child should lie upon his side, to prevent the urine from coming in contact with, and irritating the neck of the bladder. The internal remedies, from which Professor Gross has derived most benefit in the treatment of this affection, are strychnia and cantharides; of the latter he prefers the powder to the tincture, and occasionally continues the exhibition of it until slight strangury is induced. He often combines with these articles a minute portion of morphia, and, in atonic cases, some of the preparations of iron. Benzoic acid has been highly recommended, but the trials he has made with it have disappointed his expectations. Belladonna, which owes its reputation in the treatment of this affection to the recommendation of Dr. Trousseau and Dr. Blanche, of Paris, he has found more reliable. A steady persistence in the treatment for several months is necessary to insure a cure. When the morbid sensibility of the bladder is connected with inflammation, the balsam of copaiba, in moderate doses, and some full anodyne at night, especially in the form of Dover's powder, often exert a happy effect in controlling the discharge. As auxiliary measures, the cold shower-bath should be used once or twice a day, or cold water poured from a considerable height upon the lower portion of the spine, and blisters applied to the sacro-lumbar region, the perineum, or the inside of the thighs. In obstinate cases, the neck of the bladder is cauterized, as in spermatorrhœa, but much more mildly, on account of the more tender age of the patient. In the female, the application is made to the orifice of the urethra. Sometimes great benefit results from the use of anodyne enemata and suppositories. The application of pressure to the urethra, gentle but steady, and gradually increased, has sometimes been found beneficial in removing this complaint. When the incontinence depends upon the morbid sensibility of the urethra and neck of the bladder, such a proceeding is worthy of trial, especially where the more ordinary means have failed. When the tender surface is situated behind the scrotum, the probability is that the pressure of a truss, resting upon the perineum, might be serviceable. The pad should be placed directly over the middle line, and should bear so firmly upon the parts as to occlude the urethra. Tying up the penis, of course, is a proceeding which no sensible man ever would think of at the present day. That the application of the actual cautery to the sacro-lumbar region is worthy of trial in obstinate cases also of this form of incontinence, the experience of Professor Gross, in the cases reported above, seems to prove.

Of the morbid conditions of the bladder which may give rise to incontinence, *inflammation* deserves a share of attention. It may depend upon various causes, as external violence, the extension of gonorrhœa, stone in the bladder, and stricture of the urethra. The escape is usually partial, and is almost constantly associated with spasm.

The treatment consists in removing the exciting cause, which is frequently of itself sufficient to effect a cure, and in the employment of the lancet, the hip-bath, antispasmodics, and anodyne injections. The catheter often affords instant relief.

Of incontinence from *external injury* the best example is afforded in lithotomy. A kick, blow, or fall upon the perineum is occasionally followed by a similar result. Incontinence from this cause often disappears spontaneously; and, on the other hand, it is occasionally incurable. The treatment must be conducted upon general principles.

Incontinence, like retention of urine, is occasionally of a *periodical* nature, resembling, in this respect, an attack of intermittent fever, only that it is not preceded by chills, or followed by sweats. One of the best marked examples of this variety of the affection came under the observation of Professor Gross in 1849, in a young man, twenty-two years of age, a bar-keeper, of sanguine temperament, and perfectly regular habits, who, after having retired one evening, in his usual health, was seized, while asleep, with a discharge of urine, which caused him to wet his bed, and which returned afterwards, with great regularity, every night, at a certain hour. He had never suffered in this way before, and he could assign no reason for the attack. His general health was excellent, his urine normal; he was free from pain, but complained occasionally of a sense of weight and uneasiness in the neck of the bladder. When the patient applied for relief, the affection had existed for a fortnight. Professor Gross put him on the use of quinine, of which he took seven grains combined with the eighth of a grain of sulphate of morphia, every eight hours; the treatment was followed by prompt and permanent relief. A very instructive case of this form of incontinence, associated with inflammation of the neck of the bladder, and unequivocally dependent upon a miasmatic cause, is related by the late Dr. William M. Boling, of Montgomery, Alabama, in the *American Journal of the Medical Sciences* for July, 1844.

When the incontinence is irremediable, the patient should wear a urinal, to prevent the fluid from soiling his clothes. The best contrivance for this purpose is a gum-elastic bottle, shaped somewhat like a Florence flask, capable of holding about twelve ounces, and furnished at its inferior extremity with a screw for the purpose of evacuating the urine after it has accumulated to some extent in the artificial reservoir.

II. *Case of Abscess of the Prostate Gland.**—Thomas A., aged forty-eight years, of intemperate habits, entered the surgical wards of the Philadelphia Hospital on the 3d of March, 1860, on account of some trouble of the urinary organs. He was robust, and otherwise healthy, not having had, according to his declaration, any sickness since childhood, except an attack of gonorrhœa twenty years ago. At the time of his entrance he complained of not being able to evacuate the contents of his bladder, it having been necessary to introduce a catheter occasionally for that purpose. The following are the facts connected

* For notes on the history of this case, and of case IV., we are indebted to Dr. John W. Lodge, resident surgeon of the Philadelphia Hospital.

with the history of the case: During the spring of 1859, one year ago, while working in the water of the fisheries of the Delaware, the patient first noticed some obstruction to the passage of his urine, with itching at the head of his penis, and a more or less frequent desire to empty his bladder. Thus he continued during the summer and autumn, the inconvenience not being sufficient to cause him to quit his labors; the only progress the disease appeared to make was a very gradual diminution of the stream of urine, he having to wait some time for the flow to commence. The volume of the stream had so diminished when he came into the hospital, that it was with great difficulty that the bladder was emptied. On the sixth of March, three days after his admission, he had an attack of retention, for the relief of which the catheter was introduced and the urine removed. On the eighth it was necessary to use the instrument again, and, while at the prostatic portion of the canal, it came in contact with a yielding obstruction, but a little force being used, it passed into the bladder. The urine having been removed, the instrument was withdrawn to where the obstruction was felt, when from six to eight ounces of thick, cream-like pus passed through it. The instrument was inserted as far as the abscess twice in twenty-four hours during the next week or ten days, always with the effect of removing an ounce or two of pus. At the place where the abscess had broken, a cavity could be distinctly felt with the point of the instrument. The discharge gradually diminished in amount until the end of two weeks, when it had nearly ceased. The urine of the patient, examined soon after his admission, was slightly acid; some days after the opening of the abscess it was phosphatic, alkaline, and cloudy, and deposited some muco-purulent matter on standing. After the evacuation of the abscess the bladder was readily emptied by its own effort, the stream being of normal volume; the only symptom the patient complained of was some pain while the urine was passing over the site of the affection, which, however, ceased about three weeks afterwards.

In connection with this case it must, finally, be stated that the patient never suffered from symptoms of acute prostatitis. According to his own account he had no pain in the perineum, at any time, nor any tenesmus, his bowels having always been regular. Defecation was easy, and there was no fever, nor any other serious disturbance of his health.

*Clinical Remarks.*—In presenting the case reported above to his class, Professor Gross made the following remarks: The formation of abscess of the prostate is not always announced by characteristic phenomena, and hence it not unfrequently happens that the first intimation which the patient and his attendant have of the real nature of the case is a sudden discharge of pus along the urethra, consequent upon the introduction of the catheter or a violent effort at micturition. In general, however, the formation of matter is announced by well-marked local and constitutional symptoms. There is exceedingly violent pain, which assumes an aching, throbbing character; there is a sense of weight and pressure at the neck of the bladder; the patient has almost a constant desire to void his urine, which is discharged with much difficulty, and either in drops or in a small and feeble stream; the urethra is the seat of a scalding or burning sensation; the rectum feels as if it were distended by a foreign body; and more or less uneasiness is experienced in all the associated organs. In some instances the local suffering is of the most agonizing description, depriving the patient of appetite and sleep, and rapidly undermining the vital powers. Complete retention of the urine occasionally supervenes. Along with these symptoms there are generally severe rigors, alternating with flushes of heat, intense thirst, excessive restlessness, high fever, and even delirium. When this combination of phenomena exists, there can hardly be any doubt about the nature of the case, especially if the individual has previously labored under acute or chronic prostatitis. An examination by the rectum will afford additional light, and will often detect fluctuation, more particularly if the matter occupies the posterior part of the gland. At an advanced stage of the complaint, the abscess may point in the bowel or in the perineum, and thus remove all doubt respecting the diagnosis.

Any part of the prostate may become the *seat* of abscess. The middle lobe, however, is less liable to suffer than the rest of the organ, and often escapes entirely, even when the latter is nearly destroyed by it. The matter may be seated upon the surface of the gland, immediately beneath its fibrous capsule, in its proper parenchymatous structure, or in its excretory ducts. Occasionally it exists simultaneously at all these points. Abscesses of the prostate vary much both in their *number* and *size*. Sometimes, and this is what happens most frequently, there is only one, while at other times there are as many as from six to twenty, scattered through the substance of the organ, and giving it, when their contents are removed, a riddled, sieve-like appearance, not unlike that of the cribriform lamella of the ethmoid bone. Under such circumstances it is not unusual for several of them to communicate together. When numerous, their dimensions are generally proportionally small, not exceeding, perhaps, the volume of a millet-seed or a pea. Cases, in which a solitary abscess of unusually large size existed, are reported by Sir Benjamin Brodie, and by J. L. Petit, (*Œuvres Chirurgicales*, t. iii.) When the abscess is of long standing, or slow in finding an outlet, it is generally, no matter what may be its size, surrounded by a cyst of a pale, yellowish color, smooth internally, rough and flocculent on the outside, dense in texture, and from the fourth of a line to a line in thickness. The contents of such a depot do not differ essentially from those of a common phlegmonous abscess in other parts of the body. In general, they are of a light straw color, and of a thick, cream-like consistence, free from odor, and possessed of all the properties of laudable pus. Sometimes, however, they are more or less bloody, or sero-sanguinolent, and intermixed with lymph, mucus, and the debris of the affected gland. Occasionally, especially when it is long retained, the matter is excessively fetid. The structures around the abscess are infiltrated with serous and other fluids, more or less softened, and of a brownish or reddish appearance, from the injected condition of their capillaries. When the purulent depots are numerous, they are sometimes entirely disorganized, and converted into a substance closely resembling tow. A common and almost a necessary effect of abscess of the prostate is the formation of a cavity, which is often more serious in its consequences than the abscess itself.

Abscesses of the prostate open in different directions, as the urethra and the bladder, the rectum, the perineum, and the peritoneal cavity. The most natural, though at the same time the most unfortunate direction, as it respects the affected structures, in which the collection opens, is into the urinary bladder, or the orifice of the urethra, from which the matter is subsequently discharged along with the urine. Sometimes the abscess points and breaks almost simultaneously at both these situations. When it is bulky, a large quantity of pus may thus be evacuated at once; or it may drain off slowly and almost imperceptibly. In the former case the matter may be discharged in a pure state, or it may be mixed with the urine, which will then be of a lactescent, whitish, or grayish appearance, and perhaps more or less offensive; in the latter, the urine will exhibit little if any change, and deposit merely a thin, whitish sediment, visible at the bottom of the receiver. The matter may be evacuated into the rectum, and be discharged either alone or in union with the fæces. This mode of communication is by no means uncommon, and is almost certain to occur when the abscess is developed in the posterior part of the gland. The abnormal opening, situated at a variable height from the anal outlet, is generally within reach of the finger, and often continues fistulous a long time, permitting a ready interchange of the contents of the two reservoirs. The disease, in this case, is frequently complicated with inflammation and suppuration of the seminal vesicles and the adjacent structures. In the third place, the pus may escape externally by inducing ulceration of the structures of the perineum. The progress of the fluid is indicated by excessive pain in the part, and by a hard, red, circumscribed swelling, which finally points and breaks. In some instances, the matter escapes into the surrounding cellular tissue, and extends upward to the scrotum and even to the penis, following the same course as the urine when it is infiltrated into the perineum. Finally, the abscess may burst into the peritoneal cavity, at the side or posterior part of the prostate, and so cause fatal inflammation. The occurrence, which is fortunately very rare, is announced by severe pain in the pelvic region, a small, quick, and contracted pulse, violent rigors, and rapid prostration of the vital powers. Death usually occurs in from thirty-six to forty-eight hours.

Such are the various points at which the matter of a prostatic abscess may ultimately find an outlet. Of these the first is, as previously stated, the most natural as well as the most frequent, but also at the same time the most undesirable one, as it involves a greater amount of risk to the patient, from the contact of the urine with the cavity of the purulent depot after the escape of its contents. In this way an additional cause of inflammation is produced, which often operates to the destruction both of the part and the system. The passage of the matter across the perineum is uncommon, though the contrary has been asserted by Sir Benjamin Brodie, and is always attended with great delay and immense suffering, on account of the resistance offered by the fasciæ and muscles in this region. The escape of the pus through the rectum is unfortunate, as it frequently entails an obstinate fistule; but the most disastrous route of all is that in which the contents of the abscess pass into the peritoneal cavity, and excite inflammation.

Abscess of the prostate occurs at all periods of life, though not with equal frequency. Young men and adults are most prone to it; on the contrary, it is very rare in childhood and old age. Mr. Mayo records, in his *Outlines of Human Pathology*, an interesting case of it in an infant of two years. The abscess was of large size, and communicated by a considerable orifice with the urethra. The exciting causes of the disease are the same as those of inflammation of the prostate, such as gonorrhœa, stricture of the urethra, venereal excesses, external injury, and suppression of the cutaneous perspiration. It is not known what influence, if any, is exerted upon the production of this complaint by occupation, season, climate, and other circumstances. It is supposed by many, and not without reason, that chronic enlargement of the prostate, and the arthritic diathesis powerfully predispose to its occurrence.

Abscesses of the prostate are sometimes of a scrofulous nature. Professor Gross observed an instance of this kind, in a lad about fourteen years old, a subject in the dissecting-room. He was very tall and slender for his age, and the abscess, which was less than a grain of coffee, occupied the posterior extremity of the left lateral lobe, and was filled with softened tubercular matter. The gland was in other respects perfectly healthy. No history of the case could be obtained. Scrofulous abscesses are necessarily chronic and insidious in their character.

Abscess of the prostate is generally to be regarded as a dangerous affection. The local suffering, if not promptly subdued by a natural or artificial outlet for the pent-up fluid, is of itself sufficient, in many cases, to bring on serious, if not fatal, exhaustion. It behooves us, therefore, to be always guarded in our prognosis. Even under the most favorable circumstances, and where there is apparently little danger from the immediate ravages of the malady, the patient may fall a victim to its secondary effects. One of the worst consequences of this affection is a fistulous communication with the rectum, the urethra, the perineum or urinary bladder, which it is sometimes impossible to heal, and which renders the individual alike uncomfortable to himself and disagreeable to those around him. A large abscess is, of course, all other circumstances being equal, more dangerous than a small one, and a number of small ones than a solitary small one. The prognosis, moreover, will be materially influenced by the patient's habits, his age, and his previous health.

In the *treatment* of this affection, two important indications are presented: first, to limit the suppuration; and, secondly, to afford a speedy outlet to the effused fluid. To fulfill the first, prompt recourse must be had to depletion, provided this has not already been carried sufficiently far, to antimonials, diaphoretics, anodynes, and emollient applications. Leeches should be applied to the perineum and hypogastrium. In order to fulfill the second indication, the rule is to anticipate nature by an artificial opening. If the abscess point toward the perineum, an incision should be made in the most prominent part of the swelling, with a long, straight, narrow-pointed bistoury, care being taken to avoid, on the one hand, the rectum, and, on the other, the bladder. When the abscess points in the rectum, it may readily be reached with a long, curved trocar. For some days after the operation the lower bowel should be kept as quiescent as possible. When the abscess bulges inward toward the urethra and the neck of the bladder, it may be punctured with a common silver catheter; or, instead of this, a sound with a conical beak and a small curve may be used. When the abscess is not yet completely matured, and delay would be improper, the operation may be executed with the lanceted stylet. The urine should be frequently drawn off with the catheter, to prevent its entrance and sojourn in the interior of the sac. When the abscess is of a scrofulous character, as indicated by the nature of the pus, the system should be subjected to the influence of iodine and tonics.

III. *Stone in the Bladder; Lateral Operation; Unusually Large Calculus; Cure.**—James Curly, aged thirty years, a native of Ireland, was admitted into the surgical wards of the Philadelphia Hospital, on the 25th of January, 1860. According to his statement, he always enjoyed perfect health until about four years ago. There has been no hereditary taint in his family, and he himself has never had any disease of a specific nature.

In the winter of 1855–6 he enlisted in the U. S. Army, and on his voyage to Texas with his company he had a severe fall on the deck of the vessel, to which he imputes the origin of his trouble. He fell upon his back, bruising himself so severely that he was obliged to be carried down the stairs. A slight fever followed, but not enough to prevent his being present daily at the roll-call. Two weeks after the accident, the fever suddenly increased, and he experienced much pain in voiding his urine, which became thick and bloody. In spite of the medical treatment he received, he grew gradually worse; and when he joined his regiment, five or six months after his fall, the pain in his loins and along the penis was so great, in making water, that he could only do so bent over on his knees; at the same time he suffered from great tenderness in the perineum, and from a constant desire to pass water, causing him to rise often twenty times in one night. He was now subjected to repeated cupping, which had the effect of diminishing the quantity of blood voided with his urine, but did not work any change in the other symptoms. Being totally incapacitated for service, he was discharged in August, 1857. He subsequently passed through the hands of several physicians, who treated him with varying success, the only very marked improvement he experienced being from a course of nitric acid in conjunction with anodynes. In the early part of December, 1859, while working with a farmer, he was very much exposed to cold and wet, and his feet having been at one time severely chilled, he grew rapidly worse, being obliged to micturate every three or four minutes, and suffering from so intense a pain that "he had to stand on his toes and go dancing around the yard." The urine was now of varying color, but never bloody. In January, 1860, he came to this city, and having exhausted his means on divers prescriptions, applied for admission into the hospital. The severity of his symptoms had in the mean time increased, and his urine was constantly running from him. On examining the patient, Professor Gross ascertained the presence of stone in the bladder, and concluded to remove it by an operation. The man was subjected, for a

* For notes on the history of this case, we are indebted to Mr. T. C. Brainerd, a student of the Jefferson College.

few days, to a preparatory treatment, consisting of rest, abstinence from animal food, and the administration of aperient medicine.

The operation was performed on the seventh of February, before a large class of medical students. The interest of the occasion was much increased by the presence of Dr. Morton, of Boston, who administered the ether, prefacing it, at the request of Professor Gross, with an account of his discovery of this anæsthetic agent, and its first public trial in the Massachusetts General Hospital. Professor Gross, assisted by his colleagues, Drs. Agnew, Levis, and Kenderdine, made the lateral section, the only instrument used for the incision being a sharp-pointed bistoury. Two stones were found in the bladder, one of which, about an inch and a half in diameter, was removed entire; the other which was very much larger, broke into innumerable fragments, which were taken out principally with the scoop. The weight of the whole mass amounted to upwards of six ounces. It consisted principally of lithic acid. After the removal of the stones, the bladder was washed out with water from a large syringe. Owing to the very vascular condition of the parts concerned the hemorrhage was considerable. Persistent efforts were made for some time to control it with ligatures, but in vain; recourse was then had to the tampon.

On recovering from the anæsthesia, the patient "felt at once that his trouble was gone." He slept well on the ensuing night, and his bowels were moved naturally and freely four days afterward. At the end of three weeks, the urine first appeared by the natural passage, but did not cease to flow by the wound entirely until the thirteenth of April, by which time the perineum was almost completely cicatrized. The presence of the tampon had evidently greatly retarded the healing of the incision. Not a drop of blood appeared after the tampon was removed.

To improve his general health the patient is at present taking the tincture of the chloride of iron, twenty-five drops three times daily. He feels well; better than he has for four years.

IV. *Stricture of the Urethra; Urinary Abscess; Pyæmia; Death.*—John G., twenty-two years of age, was admitted into the surgical department of the Philadelphia Hospital, on the 28th of January, 1860, suffering with a rather tight stricture of the urethra; otherwise he was perfectly healthy, there being no symptom of any peculiar diathesis; his general health had always been good. From the patient the following history of his case was elicited:—

About one year ago he had an attack of gonorrhœa, which, after several weeks of treatment, was, as he supposed, cured; but, after the lapse of two weeks the discharge again began, following a protracted debauch.

By the advice of a physician he recommenced his former mode of treatment, which consisted in the use of the copaiba mixture, and injections of sulphate of copper. After the reappearance of the gonorrhœa, there was considerable pain and difficulty in voiding the urine, and acute pain immediately after the injection. Thus he continued with occasional amelioration and relapses until about six months ago, when the discharge and acute pain suddenly disappeared, the difficulty in passing the urine, together with a scalding in the posterior portion of the urethra, still remaining. From this period until his admission into the hospital the symptoms gradually increased, the time required to empty the bladder being considerable, and the urine dribbling away in a thin, spiral stream. The patient being anxious for relief, he was, on the eighth of February, influenced by chloroform with a view of effecting a cure by rapid dilatation. A small-sized metallic bougie was, without difficulty, passed into the bladder; this being immediately withdrawn, one of medium size was introduced with the employment of a little force at the site of the obstruction. After being allowed to remain for a few minutes, it was removed and replaced by a No. 7 silver catheter; this was retained by the double T-bandage, and the patient sent back to his bed. The hemorrhage which followed the introduction of the instruments did not amount to more than one or two drachms. After the operation the patient complained of slight pain and uneasiness in the region of the stricture. On the tenth, two days after the operation, the catheter was removed, cleansed, and readily replaced into the bladder. The patient continued comparatively well, passing his nights comfortably, and having no constitutional symptoms. On the fifteenth of February, seven days after the first introduction of the bougie, he was sent to the amphitheatre for the purpose of having a large instrument introduced, in continuation of the operation of dilatation; there being no suitable one at hand, the patient was carried back to his ward with the directions to have his instrument cleaned and replaced, which was accordingly done. For the three or four succeeding days he continued doing well, until about the twentieth of February, when he complained of a sharp pain in the urethra, and intense itching about the head of the penis. Warm water, medicated with Goulard's extract and opium, was applied with some advantage; the catheter being carefully examined, was found to be held in its proper place. Twenty-four hours after this, the pain in the parts became extremely severe; the patient insisting upon the removal of the catheter, it was finally withdrawn. The system was now very much affected, his skin being dry and harsh, and the pulse frequent, feeble, and irritable, with failure of appetite and rest, and a considerable discharge of unhealthy pus from the urethra. The patient was at once placed upon a suitable plan of treatment. In a day or two the scrotum became red and painful, and enlarged rapidly until fluctuation was detected, when it was opened. The incision was followed by the discharge of eight or ten ounces of urine, mixed with intensely fetid pus. Through the opening thus made, all the urine was afterwards evacuated. Abscesses were now developed in the supra-clavicular fossa, in the belly of the biceps muscle, and at one or two other points on the surface; they were successively opened, with manifest relief to the general symptoms. As soon as the case began to assume an alarming character, the patient was placed upon the use of tonics and stimulants, such as the tincture of the chloride of iron, alternated with carbonate of ammonia, milk-punch, and anodynes. But in spite of the supporting treatment, he continued to decline; the pulse became shattered and gaseous; the skin being covered with a cold, clammy sweat; and death occurred on the seventeenth of March, with all the symptoms of extreme prostration.

On examining the body after death a large abscess was found at the posterior extremity of the bladder, in the cellular tissue beneath the peritoneum where the membrane was reflected from the bladder to the rectum. The bladder itself was perfectly sound, but its cavity was empty and much contracted. A fistulous track extended from the right side of the membranous portion of the urethra, the former site of the stricture, through the perineum into the scrotum, and thence into the right groin. Along this route all the urine had been discharged. Both kidneys were remarkably congested, and occupied by an immense number of abscesses, varying from the size of a pin's head to that of a very small pea. They were equally scattered both through the tubular and vascular structures, and quite a number were seated just beneath the fibrous capsule.

The large abscess on the left side of the neck was situated beneath the collar-bone, which was almost completely deprived of periosteum, and matter had also formed in the sterno-clavicular articulation, the cartilage of which had entirely disappeared, leaving the opposed surfaces rough and discolored.

*Clinical Remarks.*—Professor Gross said one of the most serious effects occasionally witnessed, as the result of operations upon the urinary organs, especially the urethra and the neck of the bladder, is *pyæmia*, or the formation of matter in the joints, muscles, veins, cellular tissue, kidneys, liver, lungs, and other structures. The patient is seized with rigors, which, after having continued for a variable period, are followed by profuse sweats and a sense of excessive prostration. The disease, in fact, at its commencement, frequently resembles an attack of ordinary intermittent fever, the paroxysm sometimes recurring with the same degree of regularity once or twice in the twenty-four hours. Occasionally, again, it closely simulates an attack of gout or rheumatism, especially when there is intense pain in the joints and limbs. In whatever manner it makes its appearance, the case soon assumes a most threatening character. The pulse becomes small and frequent, the appetite declines, the tongue is covered with a brownish fur, the stomach is irritable, the bowels are costive, the urine is scanty and high colored, and there is excessive thirst, with constant restlessness and great anxiety of mind. Delirium and stupor generally set in at an early period, and constitute prominent phenomena of the complaint. The symptoms now described may come on within a few hours after the operation, of which they are the consequence; but, in general, they do not show themselves under two, three, or four days—at all events, not with any degree of severity. They soon assume a typhoid character, and few patients survive beyond a week, ten days, or a fortnight. The formation of matter in the external structures is usually preceded and accompanied by an erysipelatous blush of the skin, by exquisite tenderness of the part, and by great impediment in motion. The pus, which often exists in considerable quantities, either as a simple collection or in the form of distinct abscesses, is commonly of a sanious and unhealthy character, presenting the aspect rather of imperfectly elaborated lymph than of genuine matter; in some instances, it is highly fetid. The structures which are most liable to suffer are the joints, as the knee, ankle, hip, and shoulder, the muscles and cellular tissue of the extremities, the perineum and scrotum, the cellular substance and veins of the pelvis, the liver, lungs, spleen, and kidneys. The number of abscesses is sometimes very great, and, when this is the case, they are always proportionably small.

Considering the subject of pyæmia only in regard to its occurrence after operations on the urinary organs, we refrain from discussing the different theories advanced on the pathology of the disease. The most plausible one, perhaps, is that which attempts to account for the formation of purulent deposits by supposing that they are caused by the development of a peculiar poison, derived from certain kinds of fluids, purulent or other, which, entering the circulation, contaminate the blood and solids, and thus bring about that adynamic and atonic state of the system so characteristic of pyæmia. It is not improbable that this poisonous matter, whatever may be its nature, soon after its admission into the circulation, excites inflammation in the capillary vessels, as well as in some of the larger veins, rapidly followed by deposits of fibrin and pus, or the development of purulent collections. The coagula, so often met with in the veins of those who die of this disease, are a direct result of this inflammation, their formation being favored by the plasma thrown out by the inner surface of the affected vessels. Thus, it is not unlikely that the first link in the chain of morbid action in this affection is a poisoned and disorganized state of the blood; the second, the development of inflammation in the capillaries and veins; the third, the formation of adherent coagula, clots, or concretions; and the last, as the necessary and inevitable effect of the others, a deposition of pus, or pus and fibrin.

In treating of the injurious effects of operations on the urethra, we must mention another class of cases in which the symptoms produced are principally of a *nervous* character. It is well known that patients, especially such as are very nervous and irritable, occasionally suffer most violently from the most trifling operations upon the urinary organs, the mere passage of a bougie, sound, or catheter inducing violent rigors, excessive prostration, and other unpleasant symptoms. Indeed, quite a number of cases are upon record, in which death was produced by this cause, even when there was no severe disease. M. Velpeau relates, in his *Leçons Orales de Clinique Chirurgicale*, a case in which the passage of a bougie was followed, the day after the operation, by symptoms of tetanus. The patient had a violent chill the evening after the introduction of the instrument, which, as the stricture was very slight, was effected without difficulty; and he expired twenty-four hours after the appearance of the first signs of the disease. At the autopsy, the urinary organs were found to be perfectly healthy, and nothing whatever could be discovered to account for the fatal termination.

If we consider how great the natural sensibility of the urethra is, it is not surprising that the contact of a bougie, however slight, should occasionally be followed by great pain in the part, nervous prostration, and other disagreeable effects. Fortunately, all persons are not constituted alike in this particular, otherwise these effects would be of much more frequent occurrence than they are found to be in practice. The treatment of stricture, however, is peculiarly liable to be attended with pain and other unpleasant symptoms, owing to the fact that many, if not most of the subjects of this disease, are remarkably prone to derangement of the general health, and, therefore, easily affected by the most trivial operations performed for its relief.

In regard to the treatment of these nervous symptoms, much may be done to prevent them in cases where their occurrence is apprehended, by the use of chloroform; but where they are unavoidable, no time should be lost in moderating and relieving them. Promptness of action is here, indeed, of the greatest importance, for upon it often depends the safety of the patient. The treatment is plain and simple. From one to two grains of morphia, according to the age and condition of the patient, are given at a single dose, along with a liberal quantity of brandy, or brandy and spirits of camphor—a combination which is peculiarly soothing both to the part and system—the extremities and even the spine are covered with sinapisms, and cloths, wrung out of hot water and laudanum, are steadily maintained upon the genitals, the perineum, and the hypogastrium. If undue reaction takes place, abatement may be sought with the lancet and tartar emetic, or calomel and ipecacuanha; but these remedies must be employed with great caution, otherwise they may induce injurious debility.

Arthritic symptoms, and the formation of matter in the cellular tissue, joints, veins, muscles, and viscera, must be met by leeches, blisters, iodine, and warm fomentations, medicated with laudanum and acetate of lead, and by the internal use of calomel and opium, aided, if necessary, by suitable stimulants, as carbonate of ammonia, quinia, wine, brandy, and porter. Superficial abscesses must be opened by early and free incisions, both to relieve pain and to prevent further contamination of the system. But, unfortunately, as already intimated, no mode of treatment, however early or judiciously employed, can avail much under such circumstances, death being the lot of almost every patient thus affected.

V. *Case of Senile Gangrene.*—Benjamin S., a colored man, seventy years of age, was admitted into the surgical wards of the Philadelphia Hospital, on the 12th of January, with mortification of the large and small toe of the left foot. The patient had kept a stand for the sale of oysters in the street, and had been exposed to cold weather for several weeks past. When, therefore, about a fortnight before his admission, he commenced to feel a sharp, burning pain in the affected toes, he thought that it was owing to frost-bite, and applied some popular remedies for the cure of that affection; finding no relief, he entered the institution. At the time of his admission, the affected toes were found to be perfectly numb, cold, shriveled, and black; the parts adjoining them were slightly swelled, but otherwise natural; the patient complained of an acute, stinging pain in the affected parts, and of a sense of weakness. On examining the pulse at the wrist, it was found that the radial artery was completely ossified, and did not convey the least impression of any vibration to the finger. The general health of the patient had hardly suffered, his appetite was tolerably good, his bowels regular; the action of his heart was slightly increased, but otherwise natural; and he was altogether in a condition apparently justifying operative interference. The affected toes were amputated through the sound parts, and the wound was dressed with a poultice; two days afterwards, however, the stitches began to excite ulceration; erysipelas developed itself, and spread upward along the tibia; an abscess formed on the inner side of the leg, at the commencement of its lower third; on opening it, about an ounce of unhealthy pus was discharged. The patient was put on the use of tincture of chloride of iron and sulphate of quinia, and a lotion containing sugar of lead and laudanum was applied to the parts. Under this treatment, the erysipelas subsided in about a week; the wound, however, assumed a foul appearance, turned black, and discharged an extremely fetid, ichorous matter; the acute, burning pain spread over the adjoining parts, and in about two weeks after the amputation signs of mortification commenced to show themselves in the remaining toes. The suffering of the patient was now intense; his appetite failed, his tongue became coated and brown, his bladder was irritable, the urine scanty and high colored, and the action of the heart feeble and quick. Stimulants, such as milk-punch and carbonate of ammonia, were now administered alternately with the tonics; the sloughing parts were dressed with the nitric acid lotion and a yeast poultice, and a grain of morphia was given every night. Within a few days the toes became completely black, shriveled, cold, and insensible, and dropped off one after another, leaving a foul, sloughing stump, no line of demarkation having been formed. In spite of a very supportive treatment, the strength of the patient continued to decline; he became delirious, comatose, and is now, six weeks after his admission, in a dying condition.

*Clinical Remarks.*—The case under consideration, remarked Prof. Gross, is evidently one of senile gangrene, a form of chronic mortification which is most common in elderly persons. It is the same disease which was so admirably portrayed, for the first time, by Percival Pott, of London, under the appellation of mortification of the "toes and feet," and which, for this reason, was formerly known by this name. The *cause* of this disease was not, until recently, at all understood. Mr. Cowper, the anatomist, had, it is true, advanced the idea, now become general, that it was owing to ossification of the arteries, but his researches had not been conducted upon a sufficiently extensive scale to justify the positive conclusions which modern observation has so fully established. The result of the dissections made by Professor Gross is decidedly in favor of this view. Ossification of the arteries, however, is merely a predisposing, and not the immediate cause of the lesion, which consists in the formation of fibrinous clots closing up the calibre of the arteries, and thus mechanically intercepting the passage of the blood. Professor Gross has ascertained that the principal obstruction occasionally exists at a considerable distance from the seat of the disease. Thus, he has found the occlusion limited altogether to the femoral artery, the popliteal, or the commencement of the tibial and fibular. In most cases, however, it affects also the smaller branches. The concretions generally exist in various degrees of development, from recent coagulation of the blood to complete organization; hence, while some can be easily detached, others are firmly adherent to the sides of the vessels. What the immediate cause of these clot formations is has not been determined. It has been alleged that it is owing to the interception of the fibrin of the blood by the roughened walls of the arteries consequent upon their calcification; but the view of Professor Gross is, that it is due to an effusion of plastic matter, the result of chronic inflammation of the serous membrane, thereby favoring the adhesion of the blood and its conversion into clots.

Senile gangrene generally begins as a little bluish or purple speck, not larger, perhaps, than a mustard-seed, upon the inside of one of the small toes, which is soon succeeded by a minute vesicle, filled with a serous, ichorous, or sanguinolent fluid, and which, bursting, exposes a black surface beneath, perfectly cold and insensible. This spot gradually spreads in different directions until it involves the foot as high up, in many instances, as the ankle, or even the middle of the leg, although, in general, the patient dies long before it reaches that situation. Occasionally, the mortification begins at several toes simultaneously, or in pretty rapid succession; and Professor Gross has met with some cases where it first showed itself upon the heel and instep. However this may be, the part always exhibits a characteristic appearance; it is perfectly dry and withered, cold, insensible, odorless, or nearly so, and as black as charcoal, the limb looking as if it were unnaturally small, as, in fact, it generally is. During the progress of the mortification, especially if this be somewhat rapid, the skin has occasionally a mottled, purplish aspect, owing to the coagulation of the blood in the superficial veins.

The disease is usually preceded and accompanied by pains in the toes and foot, darting about in different directions, and liable to nocturnal exacerbations, preventing sleep, and rapidly undermining the general health. These pains, which are of a burning, scalding, or stinging character, are often referred by the patient to the effects of gout or rheumatism, particularly if he was formerly subject to attacks of that nature; they always increase with the spread of the malady, and demand the free use of anodynes for their suppression. The dependent posture commonly aggravates them, but, in a case which Professor Gross saw not long ago, with Dr. Levis, of this city, the suffering was immensely increased whenever the limb was elevated even for a few moments, and where, consequently, the patient, an old man of eighty-three, was constantly obliged, during the day as well as during the night, to let his foot hang down. In some instances, the attendant pain is extremely slight. Considerable swelling is sometimes present in the part, above the site of the mortification; and cases occur, although they are rare, in which the whole extremity is oppressed with œdema, being exquisitely sore, of a pale rose color, and pitting deeply under pressure. Well-marked constitutional symptoms attend this complaint, usually from the very first, and sometimes even before there is any local evidence of its presence. They are either of an asthenic type from the beginning, or they soon become so. The pulse is feeble and upwards of 120 in the minute, quick, sharp, and irritable. The tongue is coated with a brownish fur, dry, and more or less tremulous; the appetite is impaired; the bowels are costive; the alvine evacuations are fetid; the urine is scanty and high colored; the sleep is interrupted by pain and frightful dreams; the strength rapidly declines, and the patient gradually dies from sheer exhaustion, the period between this event and the commencement of the attack varying from six weeks to three or four months.

This form of gangrene occurs in both sexes, and probably with nearly equal frequency, although it was formerly supposed to be more common in men than in women. It is observed in all classes of individuals, the rich and the poor, the idle and the industrious, the temperate and the dissipated. Mr. Pott was of the opinion that the disease was peculiar to the old, but subsequent experience has shown that it may take place at different periods of life; and within the last fifteen years a number of cases have been reported where it occurred in children under ten years of age. It has been conjectured that a gouty and rheumatic temperament predisposes to the development of the affection, and there are numerous facts upon record which would seem to countenance such an idea. Again, it has been asserted that particular modes of life, as indolence and huge feeding, powerfully contribute to its production.

From the form of chronic mortification which we are now considering few patients recover. If occasionally one escapes, it only forms an exception to the general rule. In most cases the disease proceeds steadily, or, with occasional temporary interruptions, to a fatal termination. Now and then, when the powers of life are not too much exhausted, nature makes an effort to arrest the morbid action by the formation of a line of demarkation, and, this succeeding, a kind of amputation takes place, followed, after long suffering, by recovery. The event is denoted by the establishment of ulcerative action at the limits of the dead parts, immediately above which the surface exhibits a dusky, erysipelatous blush, very different from what usually occurs in ordinary gangrene. The sloughing process is generally attended with severe pain and the most offensive smell.

Much diversity still exists among authors respecting the proper method of treatment in chronic mortification; some favoring stimulating measures, while others are the warm and avowed advocates of depletion, just as if it were possible in a disease which exhibits such a protean character to lay down any one plan that shall be applicable to all cases. Without attempting to account for these discrepancies, we may state so much as certain, that no one method of treatment is applicable to chronic gangrene, although, as a general rule, the stimulant will be found to be the most reliable. Professor Gross has seen cases where, from the robust state of the individual, and the character of the pulse, no doubt could be entertained about the propriety of the employment, at least to a moderate extent, of antiphlogistic measures, where, indeed, even the lancet and antimony were admissible; but he is quite sure that such instances are comparatively few, and that, even in them, too much caution cannot be exercised in their use. Nine patients out of ten would be injured by this course. The symptoms are generally of a typhoid character from the very beginning of the malady, and not only so, but the disease nearly always occurs in old, worn-out subjects, or in persons who have long labored under depression of the nervous, vascular, and muscular powers, and who are therefore ill prepared to undergo such a plan of treatment with impunity. Tonics and stimulants, judiciously administered, and aided by appropriate measures, constitute the proper means in such cases. Sometimes a "masterly inactivity" is more effective than anything else, the surgeon doing little more than watching the case, and attending to his patient's diet, bowels, and secretions. But, in general, it will be found that a supporting plan of treatment is absolutely necessary to prevent the system from falling into a hopeless state of exhaustion. Quinine, carbonate of ammonia, and the tincture of the chloride of iron, with wine-whey or milk-punch, and opium, are the articles most to be relied upon, and they should be given in such doses as shall be calculated to meet the exigencies of each particular case. Locally, the best remedies are the diluted tincture of iodine, brushed very thoroughly twice a day over the whole of the affected part, and the use of the bandage, applied with moderate force, and kept constantly wet with a strong solution of opium and acetate of lead, Goulard's extract, or hydrochlorate of ammonia. Leeches are usually objectionable, as their bites are sometimes provocative of gangrene; and the same remark is applicable to punctures and incisions. By these means the inflammation of the obstructed vessels may now and then be promptly arrested, and the further extension of the mortification prevented.

When the sloughing process has commenced, the treatment must be conducted upon the same general principles as in the acute form of the malady, only that the local applications should, if possible, be still more mild and soothing. The most eligible remedies are, according to the experience of Professor Gross, the nitric acid lotion, in the proportion of from two to six drops to the ounce of mucilage of gum arabic, or the opiate cerate, for the ulcerated surface, and cloths constantly wet with a solution of chloride of lime for the dead, especially if there be much fetor. As the parts become detached they may be removed with the scissors, but this must be done with the greatest gentleness, as the slightest injury inflicted upon the living tissues is sure to be productive of mischief.

In regard to the question of amputation, it is extremely difficult to give any satisfactory statement. Professor Gross's belief, founded upon considerable experience, is, that we ought scrupulously to follow the practice long ago laid down by surgeons not to interfere until there is a well-marked line of demarkation; and indeed not even then, unless it is perfectly evident that there is sufficient strength of the system to bear the shock of the operation. He has, however, seen several cases where amputation was succeeded by the most happy results before nature had made any attempt to cast off the slough, and that, too, under circumstances apparently not at all promising as it respected the powers of the constitution. Whenever surgical interference is deemed advisable, no means should be spared to support the patient with tonics and stimulants, as upon their judicious use the chances of his recovery will, in great degree, depend. When the operation is performed prematurely, or before the system has sufficiently recovered from the exhausted condition consequent upon the gangrenous action, the disease will generally reappear within a few days after upon the stump, or death will follow from sheer prostration.

VI. *Case of Paralysis of the Inferior Straight Muscle of the Eye.*—John McG., twenty-eight years of age, entered the Philadelphia Hospital, in January, 1860. The patient, who was employed as porter in a store, states that about twelve months ago he fell down three stories through a hatch-way, and struck on his occiput. He was rendered insensible by the fall, and remained so for several weeks. In about two months he had completely recovered from the shock, and was able to walk about again, but has suffered ever since from steady pain in his temples, lightness of the head, and occasionally from pain in his occiput; in addition to these symptoms, he found that the movement of his eyes downward was impossible. On examining his eyes, it was discovered that, although he was able to move them inward, outward, and upward in every direction, he had no control over the inferior straight muscles. The paralysis affected both eyes in an equal degree. When requested to look downward, the patient bent his head upon his breast, but was unable to recognize objects at his feet, as the action of the muscles not affected by paralysis kept the axes of the eyeballs in an unchanged position, or even turned them somewhat upward. For several months after the accident, he had seen objects double, but is at present not troubled any more by diplopia. The pupil of his right eye is more dilated than the left, and responds much more readily to light; the appearance of the eyes is otherwise normal. The patient is affected with presbyopia, but can discern objects near at hand better with his right than with his left eye.

On his admission, the patient was put on the use of blue mass, compound extract of colocynth and jalap, and had blisters applied to the nape of his neck; after repeated purgation his headache diminished. He was now put on the use of minute doses of mercury three times a day, but when Professor Gross left the ward, no change had been effected in the condition of his eyes.

